# Formation, Development, and Cross-Species Interactions in Biofilms

**DOI:** 10.3389/fmicb.2021.757327

**Published:** 2022-01-04

**Authors:** Aihua Luo, Fang Wang, Degang Sun, Xueyu Liu, Bingchang Xin

**Affiliations:** ^1^Department of Stomatology, Guizhou Provincial People's Hospital, Guiyang, China; ^2^Department of Pharmacy, Qingdao Stomatological Hospital Affiliated to Qingdao University, Qingdao, China; ^3^Department of Cariology and Endodontology, Qingdao Stomatological Hospital Affiliated to Qingdao University, Qingdao, China; ^4^Central Laboratory, Qingdao Stomatological Hospital Affiliated to Qingdao University, Qingdao, China

**Keywords:** biofilm, cross-species interactions, metabolic interactions, quorum sensing, horizontal gene transfer

## Abstract

Biofilms, which are essential vectors of bacterial survival, protect microbes from antibiotics and host immune attack and are one of the leading causes that maintain drug-resistant chronic infections. In nature, compared with monomicrobial biofilms, polymicrobial biofilms composed of multispecies bacteria predominate, which means that it is significant to explore the interactions between microorganisms from different kingdoms, species, and strains. Cross-microbial interactions exist during biofilm development, either synergistically or antagonistically. Although research into cross-species biofilms remains at an early stage, in this review, the important mechanisms that are involved in biofilm formation are delineated. Then, recent studies that investigated cross-species cooperation or synergy, competition or antagonism in biofilms, and various components that mediate those interactions will be elaborated. To determine approaches that minimize the harmful effects of biofilms, it is important to understand the interactions between microbial species. The knowledge gained from these investigations has the potential to guide studies into microbial sociality in natural settings and to help in the design of new medicines and therapies to treat bacterial infections.

## Introduction

Biofilms are accumulated microbial communities attached to either natural or artificial surfaces (Khatoon et al., 2018). Surrounding this mono- or polymicrobial aggregate, extracellular polymeric substance (EPS) that consists of extracellular polysaccharides, cellular debris, DNAs, and proteins aim to improve microbial attachment and further formation of microcolonies. Within a mature bacterial biofilm, EPS accounts for 75–95% of the volume, and bacteria occupy only 5–25% of the volume (Huang et al., 2011). As a counterpart to planktonic bacteria, the formation of biofilms is an adaptation to hostile environments and is beneficial for bacterial survival and rapid growth (de la Fuente-Núñez et al., 2013). Bacteria within biofilms are more tolerant and resistant to antibiotic treatment, approximately 10- to 10,000-fold more than their free-swimming equivalents, which makes it difficult to destroy these microbes. National Institutes of Health in the U.S (NIH) have estimated that approximately 75% of human microbial infections are associated with biofilms (Miquel et al., 2016).

Several reviews have summarized the various deleterious effects of biofilms on human health (Muhammad et al., 2020; Flemming et al., 2021; Martin et al., 2021; Srinivasan et al., 2021). Typical bacterial biofilm-induced diseases include cystic fibrosis, periodontitis, infective endocarditis, and chronic wounds. Biofilms formed in chronic wounds result in prolonged inflammatory responses against infectious microbes and delayed wound healing (Donlan, 2001; Stewart and Bjarnsholt, 2020). In addition, biofilms form on the surfaces of medical devices, such as contact lenses, catheters, dental implants, and mechanical heart valves, which lead to persistent internal infections that could damage human organs and cause severe bacteremia (Khatoon et al., 2018; Stewart and Bjarnsholt, 2020). Moreover, biofilms present in food industries pose a high risk of foodborne outbreaks among consumers or workers, which endangers an individual’s health and economic development (Galié et al., 2018). Microorganisms grow in water distribution systems mainly by forming biofilms. Drinking water contaminated by pathogenic bacteria could damage human health and cause waterborne diseases (Hemdan et al., 2021). It is important to explore effective methods to control biofilm formation and dispersion of biofilms that are ubiquitous in human society.

To determine approaches that minimize the harmful effects of biofilms, it is important to understand the interactions between microbial species. During the development of biofilms, multifaceted mechanisms are involved in cross-species interactions (Donlan, 2001; Giaouris et al., 2015). To date, a lot of investigations have been conducted to reveal processes associated with microbe–microbe interactions. The association of different organisms allows the exchange of substrates, such as horizontal gene transfer (HGT) and metabolic interactions. These complex and dynamic communications enhance attachment and adhesion to surfaces, promote further biofilm dispersion, and therefore, result in higher persistence in the environment (Flemming et al., 2016; Muhammad et al., 2020). Organisms interact with each other and develop complex interactions that are competitive or cooperative. The competition between species is a well-recognized ecological force that drives microbial metabolism, diversity, and evolution. However, it was only recently that microbial cooperative activities were recognized as playing important roles in microbial physiology and ecology. Of note, these microbial interactions in biofilms profoundly affect their overall function, biomass, diversity, and pathogenesis (Li and Tian, 2016).

This review aims to provide a systemic insight into each type of interaction and delineate the different mechanisms that contribute to interactions, communications, and competitions between species. The information provided in this review could serve as a guide to study microbial sociality in natural settings and to design new medications and therapies to treat biofilm-related bacterial infections.

## Biofilm Formation and Development

**Figure 1:**
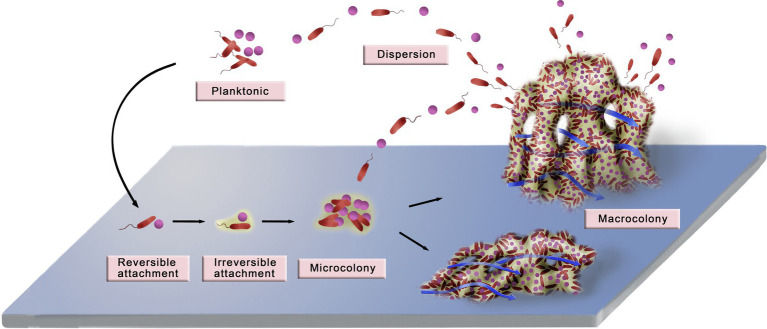
Diagram of biofilm development. The development of a biofilm can be divided into six stages: planktonic bacteria, reversible attachment, irreversible attachment, microcolony, macrocolony, and dispersion. Planktonic bacteria attach to the surface through random or active movement, and the initial attachment is unstable and reversible. Contact with the surface promotes the stable and irreversible attachment of bacteria by contact-dependent gene expression. As the planktonic bacteria continue to attach and the attached bacteria multiply, microcolonies and macrocolonies that have complex three-dimensional structures gradually form. During this process, a series of phenotypic changes occur in the compactly distributed bacteria, which make the biofilm produce new ways to adapt to the environment. The typical macrocolonies are mushroom-like protrusions that are interspersed with fluid-filled water channels. In addition, macrocolonies establish more suitable shapes to adapt better to the environment. For example, in an aquatic environment with high flow rates, a biofilm can be flat or streamlined to buffer the high fluid shear force. Finally, some bacteria detach from the microcolony and disperse into the planktonic state, which initiates another new cycle of biofilm formation. Adapted with permission from Xin et al. (2010).

In biological or natural fluids, bacteria survive in the planktonic or biofilm-inhabiting state. Living in a biofilm matrix renders microbes highly resistant to antibiotics and the host’s immune responses. Recently, it was reported that through a series of complex and dynamic processes, microorganisms develop from free-swimming individuals to congregated biofilms. Despite distinctive microbial species and extracellular microenvironments, the formation of different biofilms shares several common characteristics (Monds and O’Toole, 2009; Kostakioti et al., 2013; Rath et al., 2021). In general, a well-known model of biofilm formation that applies to Gram-positive and Gram-negative bacteria is composed of the following stages, which are shown in Figure 1.

### Reversible Attachment

The prerequisite for the formation of a biofilm is that planktonic bacteria should approach and contact a surface. During this initial step of attachment, motilities play a critical role. Brownian motion, during which particles randomly collide with each other, has been suggested to play a crucial role in the movement of non-appendaged bacteria, such as *Staphylococcus* sp. and *Klebsiella* sp. (Floyd et al., 2017). Other biological factors, such as flagella and type IV pili (TFP), have an impact on bacterial motility through fluids. Additional combinations with adhesive molecules, such as adhesive pili, proteins, and surface-bounded EPS, facilitate bacterial adhesion to a surface (Kostakioti et al., 2013; Belas, 2014; Guo et al., 2021). Despite the effective adhesins, the attachment is fragile and usually transient during this stage, because of hydrodynamics and repulsive forces at the boundary layer and the inadequate production and function of adhesive molecules (Petrova and Sauer, 2012; Kreve and Reis, 2021). Long-term attachment to the surface results in contact-dependent gene expression, which ultimately leads to the alteration from reversible to irreversible attachment.

### Irreversible Attachment

Irreversible attachment means a sessile and sedentary adhesion to the substratum. Contact-dependent signaling pathways induce the production of important mediators for stable adhesion (Petrova and Sauer, 2012). After rounds of detachment and attachment, the second messenger cAMP creates an intermediate surface sentient state between motile and sessile in the model biofilm bacteria *Pseudomonas aeruginosa*. The Pil-Chp surface-sensing system contributes to an increasing level of cAMP, which oscillates and ultimately downregulates the activity of type IV pili. Multigenerational cAMP-TFP oscillations facilitate progression to the irreversible attachment stage (Luo et al., 2015; Lee et al., 2018). Another important mediator is bis-(3′-5′)-cyclic dimeric guanosine monophosphate (c-di-GMP), an essential factor that determines the change from free-moving to the attached state for several Gram-negative bacteria (Römling et al., 2013; Liu et al., 2020). C-di-GMP promotes the production of EPS. A study into *P. aeruginosa* suggests that exopolysaccharide Psl induces intracellular synthesis of c-di-GMP, which decreases flagella and elevates the level of Psl and other EPS molecules (Irie et al., 2012). Moreover, in *P. aeruginosa*, c-di-GMP can repress the synthesis of flagella by binding with transcription factor FleQ (Baraquet and Harwood, 2013). During the irreversible attachment stage, microbial cells accumulate and aggregate in layers and produce massive EPS.

### Biofilm Maturation (Microcolony and Macrocolony)

As an increasing number of bacteria attach irreversibly and the attached bacteria multiply, biofilms gradually form with complex structures and diverse bacterial species. Then, microcolonies, the biofilm precursor, are built and further develop into matured macrocolony biofilms. On the formation of microcolonies, encapsulated bacteria produce large amounts of EPS, which supports inner microbes and shields them against antibacterial molecules. EPS components vary between different bacterial species or even strains of the same species but commonly include polysaccharides, proteins, and DNAs (Floyd et al., 2017). During cariogenic biofilm formation, the binding interactions between *Streptococcus mutans* and *Candida albicans* are selectively increased when *C. albicans* cell wall is coated with extracellular glucans. Of interest, co-existing *C. albicans* promotes *S. mutans* biofilm maturation and maintains an acidic environment, which contributes to the pathogenesis of dental caries (Kim et al., 2020). *Escherichia coli* mainly produces cellulose as the exopolysaccharide constituent and *P. aeruginosa* secretes polysaccharides that involve Psl, Pel, and alginate. However, not all *P. aeruginosa* strains produce these three types of compounds. Mucoid strains of *P. aeruginosa* are the causative agent of cystic fibrosis and excrete all three polysaccharides, of which alginate is dominant. Alginate is associated with persistence and immune evasion and promotes *P. aeruginosa* coinfection with *Staphylococcus aureus* in cystic fibrosis disease. Non-mucoid *P. aeruginosa* strains, PAO1 and PA14, only produce Psl and Pel (Wozniak et al., 2003; Irie et al., 2012; Wei and Ma, 2013; Limoli et al., 2017).

During this phase, bacteria continuously grow and proliferate with new inner cells that attach to the surface and the whole biofilm spatially expands upward. Matured biofilm is characterized by a mushroom-shaped three-dimensional formation (Muhammad et al., 2020). A recent study into biofilm-forming *Vibrio cholerae* suggested that intercellular mechanical potentials resulted in cellular order and overall construction by the production of specific EPS ingredients. In addition, shear flow, one of the external factors, plays a key role in shaping biofilm architecture by competing against cell–cell attraction forces (Hartmann et al., 2019). Mature biofilms encompass internal microbes and protect them from being killed by chemotherapies and host defenses and continue to exist for a prolonged period (Breidenstein et al., 2011).

### Dispersion

As a biofilm matures, some microorganisms detach from the biomass and disperse. These discrete bacteria could reattach into a surface and form a secondary biofilm or float freely in the intermediate environment (Kaplan, 2010). Two main mechanisms are involved in the dispersal of biofilm bacteria (Rumbaugh and Sauer, 2020; Wille and Coenye, 2020). Passive dispersion is decided by mechanical or shear stress. For example, the weight of a 67 h-old *S. mutans* biofilm biomass was reduced to only 15% of primary mass after treatment by 10 times increased shear stresses (Hwang et al., 2014). Based on this knowledge, high-pressure pulsating water is now used to clear dental plaque that causes dental decay when deposited on tooth enamel. The water jet is much more efficient than toothbrushes to eradicate dental plaque from hidden and narrow areas (Sharma et al., 2008). In contrast to passive dispersion, active biofilm dispersal is initiated by bacterial responses to triggers, such as nutrient abundance and atmospheric triggers (hypoxia or low nitric oxide), and results from the activation of several signaling factors (James et al., 1995; Jiang et al., 2020). One of the main mechanisms is the reduction of intracellular c-di-GMP. For example, accumulated nitric oxide is sensed by NbdA and activates phosphodiesterase, which degrades c-di-GMP in *P. aeruginosa*. Decreased levels of c-di-GMP are associated with elevated motility and downregulated EPS synthesis, leading to lower tackiness and ultimate dissemination (Li et al., 2013; Cutruzzolà and Frankenberg-Dinkel, 2016). Another important mechanism is the involvement of extracellular enzymes that target structural EPS ingredients. *S. aureus* secretes four types of proteases, serine protease (SspA), cysteine protease (SspB), staphopain (ScpA), and metalloprotease (Aur), which lead to the degradation of extracellular proteins (Fleming and Rumbaugh, 2017). Dispersin B, produced by several bacteria including *S. aureus, Klebsiella pneumoniae*, and *E. coli*, is a glycoside hydrolase that degrades the exopolysaccharide and displays biofilm disorganizing functions (Fleming and Rumbaugh, 2017; Guilhen et al., 2017; Wille and Coenye, 2020).

Of note, different types of substrates have great impact on the formation and development of biofilms. On artificial substrates (carbon fiber and polyvinyl chloride), bacterial network patterns are more complex than those formed on natural surfaces (pebble and wood). It is also reported that bacteria colonized on artificial substrates are more powerful in metabolizing nitrogen, carbon, and arsenic sources (Miao et al., 2021). Therefore, the effects of different substrate types on the dynamics of biofilm community could attract more attention in the field of biofilm research. In addition, modeling biofilm formation *in vitro* allows the biofilm to be studied more closely and helps us to explore potential treatments for the targeted biofilms. For instance, flow chambers are promising approaches that could be used to assess bacterial adhesion to biomaterial surfaces, which is evaluated by microscopy (Gomes and Mergulhão, 2021). Microfluidic platforms contain multiple channels to study bacterial adhesion, biofilm formation, or antimicrobial treatments. A newly designed microfluid chip, BiofilmChip, recreates polymicrobial biofilm formation under clinical conditions and monitors the heterogeneous biofilm microenvironment (Blanco-Cabra et al., 2021). To gain knowledge on biofilm formation and to test antimicrobial agents directly, investigations are required to validate existing *in vitro* platforms further and to develop more effective techniques.

## Microbe–Microbe Interactions in Biofilms

Biofilms are aggregated communities of microbes, which are not limited to bacteria and can involve eukaryotes. Fungi, such as *Candida* sp., are well-studied biofilm-building microorganisms. Theoretically, one microbial species could predominate in a biofilm, which is called a monomicrobial biofilm. However, this is unusual, and most biofilms persist in polymicrobial compositions. Cross-species or even cross-kingdom interactions occur during biofilm formation and contribute to the biofilm homeostasis in polymicrobial communities (Peters et al., 2012). To date, a diverse array of biomolecules has been identified (Table 1). For example, it is difficult for *S. aureus* alone to form monoculture biofilm; however, co-existing with *C. albicans*, which provides a supportive scaffold, allows for sustainable biofilm formation and enhanced resistance to vancomycin (Harriott and Noverr, 2009). In addition, this dual-interaction benefits *C. albicans*, which is indicated by increased resistance to antifungal miconazole (Kean et al., 2017). Despite collaborative interactions, competitive and antagonistic communications exist and influence the homeostasis or pathogenesis of biofilms (Table 1). The environment and sequence of colonization play a key role to determine the type of interactions in polymicrobial biofilms, for example, cooperation or competition, evidenced by a study on *P. aeruginosa* in cystic fibrosis and oral commensal streptococci (Whiley et al., 2015).

**Table 1:** Summary of main cross-species interactions in biofilms.

	Species involved in interaction	Interaction type	Reference
Cooperation/synergy	*F. nucleatum* and *Clostridioides difficile*	Coaggregation: adhesin RadD on *F. nucleatum* with flagella on *C. difficile*	Engevik et al., 2021
	*P. gingivalis* and *S. gordonii*	Coaggregation: Mfa1 protein on short fimbriae of *P. gingivalis* with the SspB polypeptide on *S. gordonii* surface	Park et al., 2005; Kuboniwa et al., 2006
	*S. oralis* and *P. gingivalis*	Coaggregation: glyceraldehyde-3-phosphate dehydrogenase of *S. oralis* with *P. gingivalis* fimbriae	Maeda et al., 2004
	*S. gordonii* and *V. parvula*	Coaggregation interaction involves oxidative stress-related processes and carbohydrate metabolism	Mutha et al., 2019
	29 species of bacteria and 1 fungal species were evaluated	Coaggregation often mediated by interaction between lectin and saccharide	Stevens et al., 2015
	*Acinetobacter* sp. strain C6 and P*. putida*	Metabolic interactions: *P. putida* uses benzoate from *Acinetobacter* sp. strain C6	Hansen et al., 2007
	*Streptococcus gordonii* and *Veillonella atypica*	Metabolic interactions: *S. gordonii* produces lactic acid that is used by *V. atypica*	Egland et al., 2004
	*F. nucleatum with P. gingivalis*, *T. denticola*, and *T. forsythia*	QS: AI-2 produced by *F. nucleatum* promotes the growth of *P. gingivalis*, *T. denticola*, and *T. forsythia*	Jang et al., 2013
	2 bacterial strains from soil and *P. fluorescens*	Volatiles (benzonitrile and dimethyldisulfide) produced by Collimonas pratensis and Serratia plymuthica stimulate *P. fluorescens* growth	Garbeva et al., 2014
	*Bacillus subtilis* and *P. aeruginosa*	Electrical signals generated by *B. subtilis* biofilm can attract *P. aeruginosa*	Humphries et al., 2017
Competition/antagonism	*Brevibacillus* sp. M1-5 and *Pseudoxanthomonas* sp. M1-3	Competition for oxygen The growth of facultative aerobe *Pseudoxanthomonas* sp. M1-3 suppresses the viability of *Brevibacillus* sp. M1-5	Yamamoto et al., 2010
	*K. pneumoniae* and *E. coli*	Competition for iron where K*. pneumoniae* grows better than *E. coli*	Juarez and Galván, 2018
	*S. gordonii*, *S. sanguinis*, and *S. mutans*	*S. gordonii* and *S. sanguinis* produce H_2_O_2_ to inhibit *S. mutans*	Kreth et al., 2008

## Cooperation and Synergy in Biofilms

Because polymicrobial constituents are common in nature, the synergism and cooperation between different microbes are important to maintain the coexistence of different microbial species and biofilm homeostasis, which outcompete the mutual antagonistic effects (Huang et al., 2011). Bacteria achieve this through cooperation, forming a community in which all species are located closely together. This type of cooperation could evolve if the interests of two or more parties are directly aligned (Buckling and Brockhurst, 2008). Previous studies have suggested the following mechanisms for cooperation and synergy.

### Cohesion and Coaggregation

The prerequisite to forming mixed-species biofilms is the coexistence of distinctive microbes (Li and Tian, 2016). A recent study into 29 bacteria and 1 fungus isolated from various environments demonstrated that 77% of these 30 strains coaggregated with at least another strain and 70% coaggregated with other archetypic strains. This coaggregation was mostly mediated by the interaction between lectin and saccharide (Stevens et al., 2015). To date, the most studied cohesion and coaggregation model are the oral biofilm–dental plaque. *Streptococcus gordonii*, a first colonizer on dental plaque, provides specific cues to colonize with *Porphyromonas gingivalis* into a polymicrobial biofilm. Apart from the interaction of SspB (a surface polypeptide on *S. gordonii*) with short fimbriae on *P. gingivalis*, some components identified in *S. gordonii* are associated with this interconnection, including adhesive proteins and extracellular capsules (Park et al., 2005; Kuboniwa et al., 2006). Another initial colonizer, *Streptococcus oralis*, secretes glyceraldehyde-3-phosphate dehydrogenase, which acts as a coadhesin by binding to major fimbriae on *P. gingivalis* (Maeda et al., 2004). In addition to the motility organelles involved in cohesion, to unravel the interaction between two initiators of dental plaque, *S. gordonii* and *Veillonella parvula*, dual-RNA sequences on each strain indicated that oxidative stress-associated processes dominated the *V. parvula* coaggregation responses and *S. gordonii* mainly focused on carbohydrate metabolism during this process (Mutha et al., 2019).

In summary, microbes initiate cohesion and coaggregation by producing several adhesive components and induce intercellular interactions to promote multispecies coexistence within a biofilm.

### Metabolic Interactions

After interbacterial coaggregation, which results in efficient cell-to-cell communication, the metabolic interactions emerged as another important cooperative strategy.

Different species cooperate to break down a common substrate into usable nutrients and achieve nutritional interdependence. For example, inside a multispecies biofilm consortium with *Acinetobacter johnsonii* strain C6 (Kaas et al., 2017), *Pseudomonas putida* evolves into rough colony variants by natural selection (Hansen et al., 2007). Specifically, when both bacteria originally used aromatic benzyl alcohol as their only carbon source, the *P. putida* variant was dependent on the metabolite, benzoate, from the *A. johnsonii* and could survive oxygen starvation in the mixed-species biofilm. The variant of *wapH* mutation increased the production of a cellulose-like polymer, therefore, enhancing adherence with *A. johnsonii* (Hansen et al., 2007).

However, when different strains have access to distinctive nutrient substrates, they have the potential to exchange metabolites and catalyze them further in another pathway. This cross-feeding or syntrophy is a common mechanism during metabolic interactions (Adamowicz et al., 2018; Evans et al., 2020). Using oral biofilms as an example, *Veillonella* sp. rely on lactate as a nutrient source. *Veillonella* sp. are frequently in symbiosis with lactic acid-producing bacteria, such as *S. oralis* and *S. mutans*. This type of cross-feeding on lactate has been observed between *S. gordonii* and *Aggregatibacter actinomycetemcomitans* (Guo et al., 2014; Bowen et al., 2018). In addition, *S. gordonii* expresses putative arginine-ornithine antiporter ArcD, which plays an important role in arginine uptake and maintenance of nutrient supply. Arginine is commonly used by *S. gordonii* and transformed into ATP and NH_3_, which are beneficial for bacterial growth and homeostasis. Ornithine is exported by ArcD from *S. gordonii* to *Fusobacterium nucleatum*, in which orthenine is broken down by ornithine decarboxylase into putrescine. Simultaneously, a dual-species biofilm of *F. nucleatum* and *S. gordonii* is attenuated by arcD depletion, whereas restoring ornithine reverses inhibited biomass of this mixed-species biofilm, which suggests the need for ornithine cross-feeding to maintain biofilm development (Jakubovics et al., 2008; Sakanaka et al., 2015). Despite the well-studied cross-feeding between different species, unidirectional cross-feeding in single-species biofilm has been identified. Investigations into the sputum from cystic fibrosis patients found oxic and hypoxic zones. Under anaerobic conditions, *P. aeruginosa* reduces pyruvate to lactate through lactate dehydrogenase, which is further used as a nutritional supplement. Lactate induces the active expression of IldE, the enzyme that catalyzes D-lactate oxidation during aerobic growth. Due to the spatial distribution of oxygen, lactate produced by *P. aeruginosa* in an anoxic zone further feeds the bacteria in the oxic area and activates LldE expression (Lin et al., 2018; Evans et al., 2020). In addition, *P. aeruginosa* could survive on propionate and acetate converted from mucin by commensal anaerobic bacteria, and genes involved in propionate utilization are activated during *in vivo* cystic fibrosis. *P. aeruginosa* uses mucin as another carbon source by cross-feeding from other species (Flynn et al., 2016). Cross-feeding is beneficial because it gives single or multispecies biofilm systems higher metabolic efficiency by maximally utilizing waste products and makes nutrient sources available for the microorganisms to support their growths.

### Quorum Sensing

Through quorum sensing (QS), in which small diffusible signal molecules (autoinducers) are released and are detected to induce gene expression in a coordinated manner, mono or polymicrobial communities achieve cell–cell communication in response to microbial density (Abisado et al., 2018). In general, Gram-positive bacteria produce autoinducing peptides for QS and Gram-negative bacteria use acyl-homoserine lactones as signal factors whose synthesis depends on the LuxI-like protein. Another type of QS, LuxS-encoded autoinducer 2 (AI-2), is found in Gram-negative and Gram-positive bacteria, which mediates interspecies interactions (Li and Tian, 2012; Mukherjee and Bassler, 2019). For example, AI-2 produced by *S. oralis* promotes the development of a mixed biofilm with *Actinomyces naeslundii*, which was demonstrated by the inability of *S. oralis* that had mutated LuxS to form a biofilm with *A. naeslundii* and the recovered ability to form this dual-species biofilm after restoring AI-2 synthesis (Rickard et al., 2006). In addition, *F. nucleatum* secretes AI-2 that significantly induces coaggregation with each species in a red complex (*Porphyromonas gingivalis*, *Treponema denticola*, and *Tannerella forsythia*) and this process is inhibited by QS inhibitors. Specifically, AI-2 triggers the production of bacterial surface adhesins and mediates interspecies interactions between period into pathogens (Jang et al., 2013). As in gastrointestinal niches, Gram-positive bacterium *Enterococcus faecalis* produces AI-2 that attracts *E. coli* and maintains their expression of *lsr* operon even at low cell densities, which results in enhanced aggregation of microcolonies (Laganenka and Sourjik, 2018). Apart from QS between bacterial species, interkingdom interactions *via* QS have been demonstrated. *S. gordonii* produces AI-2 that is sensed by the fungi *C. albicans* and induces elevated hyphae synthesis and modulates the expression of mitogen-activated protein kinases (Bamford et al., 2009). In addition, the concentration of signal molecules has a role. A QS molecule from *C. albicans*, farnesol, enhances growth and microcolony formation of *S. mutans* at relatively low levels, whereas a higher concentration of this molecule inhibits *S. mutans* proliferation (Kim et al., 2017).

In summary, QS plays an indispensable role in biofilm formation and interspecies or cross-kingdom communication by inducing associated gene expressions.

### Horizontal Gene Transfer

**Figure 2:**
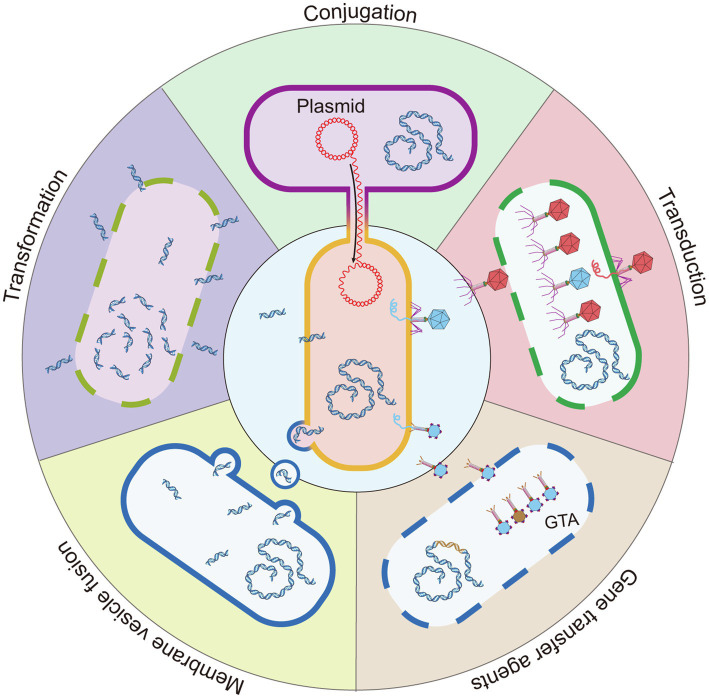
Five mechanisms for HGT. Conjugation is the HGT of bacteria by direct contact, and the DNA of the donor bacteria is transmitted to the recipient bacteria by the conjugative pili or adhesins. Transformation: the lysed bacteria (dashed line) release naked DNA fragments, which are acquired, integrated, and expressed by other bacteria. Transduction: a type of HGT mediated by bacteriophage. After the bacteriophage infects the bacteria, bacterial DNA fragments might be accidentally loaded into the bacteriophage head. The bacteriophage that carries the host bacteria’s DNA is released and infects a new bacterium to complete HGT. The bacteriophage with a red head represents that carries bacteriophage DNA and the blue head represents a bacteriophage that carries host bacterial DNA. Membrane vesicle fusion: the bacterial outer membrane bulges to form 20–250 nm MVs that carry genetic material and releases it into the environment. MVs have a lipid bilayer biological membrane that can protect and transport cargo and fuse with target cells to deliver contents. GTAs: bacteria that carry the GTA gene in chromosomes (brown fragment) can produce GTA. Most GTA particles carry a small random DNA fragment of the producing bacteria (blue particle head). A few GTA particles carry partial fragments of the GTA gene (brown particle head). Because these GTA gene fragments are not complete, they cannot be encoded into new GTA particles after being transferred into the recipient cell. GTA particles are released by bacteria cell lysis (dashed line).

Close cell–cell contact in biofilms creates a favorable condition for HGT. To date, it is well known that HGT occurs with increased competence in biofilms. Several classic mechanisms are involved in HGT, which include conjugation, transformation, and transduction (Molin and Tolker-Nielsen, 2003). In addition, recent studies have revealed other mechanisms that include membrane vesicles (MVs) and gene transfer agents (GTAs; Figure 2).

Conjugation is a direct cell-to-cell transfer by plasmids or transposons. For instance, in oral biofilm structures, live donor bacterium *Veillonella dispar* can transfer the conjugative transposon Tn916 to four different *Streptococcus* spp. that are recipients in a multispecies oral consortium that grows as a biofilm (Hannan et al., 2010). In addition, one study into soil bacteria indicated plasmid permissiveness during conjugation in a recipient-dependent manner. *E. coli* transfers the IncP1 plasmid, pKJK10, to a mixed collection of 15 bacterial strains; however, only the transfer of this plasmid from *P. putida* to *Stenotrophomonas rhizophila* succeeded, and transfer to the mixed community was not observed (de la Cruz-Perera et al., 2013).

Transformation is the simple use of eDNA in the surrounding environments. In marine biofilms on chitinous surfaces, QS in *V. cholerae* that was mediated by AI-2 and CAI-I enhanced the competence of eDNA uptake by inducing competent gene *comEA* expression when cocultured in a mixed-species biofilm (Antonova and Hammer, 2011). eDNA is often released after cell autolysis. In an experimental biofilm, the transformation of purified *V. dispar* genomic DNA to *Streptococcus mitis* gave *S. mitis* tetracycline resistance (Hannan et al., 2010).

Transduction refers to the transfer of host genomic DNA in which bacteriophages are the carriers. Bacteriophages that carry Shiga toxin (Stx)-encoding genes promoted the emergence of a new Stx-producing *E. coli* (STEC) within the biofilm at 37°C or 20°C, which suggested that a biofilm environment was suitable for transduction and promoted interactions between strains of the same species (Solheim et al., 2013).

MVs are released by bacteria and carry genetic materials that fuse with the recipient bacteria for HGT (Toyofuku et al., 2019). GTAs are phage-like particles that are produced by bacteria, which carry random DNA fragments from the original bacteria and transfer this into the recipient bacteria (Lang et al., 2012; Bárdy et al., 2020). MVs and GTAs are important factors for HGT in a marine planktonic ecological environment (Paul, 2008; Lang et al., 2012; Lossouarn et al., 2015; Biller et al., 2017). Recent research found that MVs promoted the formation of single-strain bacterial biofilms (Seike et al., 2020). The MVs from *S. mutans* augmented *C. albicans* biofilm development but had no significant effect on *C. albicans* growth under planktonic conditions (Wu et al., 2020). However, the role of MVs and GTAs in multistrain bacterial biofilms remain unclear. Further investigations into MVs and GTAs are required to expand the knowledge on HGT in biofilms.

Of interest, previous studies suggested that the density of cell populations in biofilms promoted plasmid dispersal *via* conjugation, which then induced biofilm maturation (Molin and Tolker-Nielsen, 2003; Madsen et al., 2012). In addition, released DNA enhanced the stability of biofilms by synthesizing EPS (Montanaro et al., 2011). In summary, a biofilm provides a suitable environment for HGT, which then produces populations with antibiotic resistance or higher pathogenicity, which promotes the homeostasis of the biofilm (Madsen et al., 2012).

### Production of Beneficial Components

Microorganisms in polymicrobial biofilms benefit each other by secreting certain molecules. Within polymicrobial biofilms of otitis media, *Moraxella catarrhalis* secretes β-lactamase and protects *Streptococcus pneumoniae* from death by β-lactam, and a quorum signal (AI-2) generated by *S. pneumoniae* further increases *M. catarrhalis* colonization (Perez et al., 2014). This type of positive feedback loop makes either microorganism more tolerant to clearance (Perez et al., 2014). In dental plaque biofilms, for example, interkingdom synergism has been characterized. Glucosyltransferase B, an exoenzyme derived from *S. mutans*, binds to the mannan layer of *C. albicans*, which augments EPS formation and the development of mixed-species biofilm (Ellepola et al., 2017; Hwang et al., 2017). In turn, *C. albicans* produces polysaccharides to support colonization and adhesion of *S. mutans*, which promotes mixed biofilm formation and induces dental caries (Khoury et al., 2020). Apart from mutually supportive interactions, biofilm bacteria can protect other microorganisms. *Streptococcus sanguinis* in oral cavities produces hydrogen peroxide (H_2_O_2_), which suppresses the survival of *P. gingivalis*. The presence of *A. actinomycetemcomitans* reverses this inhibition through the secretion of *KatA*-encoded catalase, which reduces H_2_O_2_ concentrations, and therefore, supports the survival of *P. gingivalis* in this tri-species biofilm (Zhu et al., 2019). Thus, when the diversity of microorganisms in a biofilm increases, mechanisms underlying the interactions between different species become more complex. To gain more knowledge about the panorama of biofilm community, we need to understand the competitive and antagonistic interactions in addition to cooperation and synergism.

## Competition and Antagonism in Biofilms

Bacterial species maintain a proper distance; therefore, they avoid strong substrate competition or toxic compounds secreted by other species (Kim et al., 2008). The production of antagonistic compounds, such as bacteriocins, H_2_O_2_, organic acids, different enzymes, and the release of lytic phages, is a few examples of weapons that could give an organism a competitive advantage over other organisms during colonization and competition (Marsh and Zaura, 2017). Research has focused on the competitive and antagonistic interactions within biofilms, which involve competition for nutrients and binding sites, the release of inhibitory signaling molecules, or lytic phages. In addition, competition in biofilms helps to maintain biofilm stability and is another autoregulatory network to maintain a balanced state within microbial communities.

### Competition for Nutrients and Public Goods

Due to the requirements for limited nutrients, different bacterial species compete for nutrients to survive. For example, obligate aerobe *Brevibacillus* sp. M1-5 primarily dominates at the air–water interface, the emergence of the facultative aerobe *Pseudoxanthomonas* sp. M1-3 competes for oxygen, which decreases the number of viable *Brevibacillus* sp. (Yamamoto et al., 2010). Another example is iron chelation that uses siderophore. In the dual-species biofilm composed of *Pseudomonas fluorescens* and *P. putida*, chelator production provides an absolute growth advantage over non-chelator populations (Eberl and Collinson, 2009). Uropathogenic strains of *K. pneumoniae* and *E. coli* compete for iron, in which *K. pneumoniae* grows better than *E. coli* by the production of siderophores. The competition for nutrients, in particular iron, ultimately modulates composition (*K. pneumoniae*: *E. coli* ratio, 55:1; Juarez and Galván, 2018). Therefore, the ability to effectively use nutrients promotes bacterial growth to the detriment of its competitors, which leads to changes in biofilm composition.

### Production of Deleterious Components

In a biofilm, several bacteria could produce antibacterial or bacteriostatic proteins, for example, bacteriocins. The main difference between bacteriocins and antibiotics is that bacteriocins show unique activity to strains that belong to the (same) producing species (García-Bayona and Comstock, 2018). For example, *Streptococcus salivarius* in the oral cavity contributes to the production of large amounts of bacteriocins. Santagati et al. identified 13 from 81 α-hemolytic streptococci strains, which were isolated from healthy children, produced bacteriocins, and inhibited different Gram-positive pathogens, such as *S. pneumoniae* (Santagati et al., 2012). Another study demonstrated the probiotic ability of *S. salivarius* 24SMB and *S. oralis* 89a, which disturbed the biofilm development or even dissolved preexisting biofilms from typical upper respiratory tract pathogens that involved *Streptococcus pyogenes*, *Streptococcus pneumoniae*, *Moraxella catarrhalis*, *S. aureus*, *Staphylococcus epidermidis*, and *Propionibacterium acnes* (Bidossi et al., 2018).

Studies have found that about 30–50% of *E. coli* strains are efficient producers of colicin (Riley and Gordon, 1992; Gordon et al., 1998; Smarda and Smajs, 1998; Gillor et al., 2004). Colicin is possibly encoded by the *kil* gene-containing plasmids and released into the extracellular environment by cell lysis (Rendueles and Ghigo, 2015). Colicins are a particular group of bacteriocins that are produced by and toxic to some strains of *E. coli* and other enteric bacteria and colicin-producing strains are resistant to the colicin they produce (Hibbing et al., 2010). By binding to surface receptors and then entering colicin-sensitive cells, colicin mediates cell death by producing pores on the membrane or degrading DNA and RNA. In response to SOS stress, colicin R is induced in *E. coli* ROAR029 that resides in a matured mixed biofilm rather than in a free-swimming status, which exerts highly competitive functions against enteroaggregative *E. coli* LF82 by pore formation (Rendueles et al., 2014).

Various bacteria produce toxic molecules that kill neighboring bacteria. A well-studied low molecular weight compound is H_2_O_2._ Studies into oral species *S. gordonii*, *S. sanguinis*, and caries-inducing *S. mutans* demonstrated the importance of H_2_O_2._ *S. gordonii* and *S. sanguinis* produce H_2_O_2_ *via* SpxB-encoded pyruvate oxidase to inhibit pathogenic *S. mutans*. This antagonism is modulated by glucose and oxygen availability. In addition, *S. mutans* produce mutacins, which have cytolytic functions on other *Streptococcus* species (Kreth et al., 2008). *A. actinomycetemcomitans* have developed responses against H_2_O_2_ by secreting catalase (*KatA*) and Dispersin B (DspB) that detoxifies H_2_O_2_ and assists bacteria to escape from damage, respectively, which suggests an adaptive mechanism to counteract disruptions by toxic compounds (Stacy et al., 2014).

## Concluding Remarks

Recent studies into biofilms have provided significant knowledge and understanding of the specific mechanisms that are involved in biofilm formation. As reviewed previously, interspecies communication and competition play a vital role in balancing microbial populations in biofilms. In addition, influenced by external or internal microenvironments, biofilms contain bacteria that either cooperate to achieve optimal survival and use of nutrients, such as *S. gordonii* and *A. actinomycetemcomitans*, or produce compounds that inhibit the proliferation of their competitors, such as *K. pneumoniae* and *E. coli*, that compete for iron. It is well known that biofilms that form on medical devices or inside specific organs cause severe chronic infections, which are resistant to chemotherapeutic treatment, and therefore, survive for a long time. Therefore, many researchers are exploring new and promising inhibitors against biofilms.

This review discussed several recognized mechanisms that mediate synergistic and antagonistic communications between microorganisms. The delicate interactions between microbes in biofilms are complicated and involve known and unknown mechanisms. Despite the increased understanding of interspecies, strains, and kingdoms interactions, some questions remain about biofilm balance and stability which affects the pace of new findings. Future research should focus on the following aspects: (1) could new antibiofilm strategies be designed based on the molecular mechanisms involved in interspecies cooperation or competition? Is it possible to develop drugs that target the mediators of microbial interactions in biofilms to inhibit biofilm maturation without killing the beneficial microorganisms and causing micro-ecological imbalance? (2) what strategies could be used to disturb biofilm homeostasis and what components are essential to maintain biofilm complexity? and (3) how could high-dimensional technologies be utilized to investigate bacterial interactions in biofilms more systemically and comprehensively? Further understanding of the molecular mechanism of species interactions at different stages of biofilm formation, development, and maturation could open a new method to address the problem of biofilms.

## Data Availability Statement

The raw data supporting the conclusions of this article will be made available by the authors, without undue reservation.

## Author Contributions

AL and BX: conception, investigation, and writing original draft. FW: resources, investigation, and writing original draft. DS: review and editing. XL: resources, investigation, and data curation. All authors contributed to the article and approved the submitted version.

## Funding

This work was sponsored by the Qingdao Key Health Discipline Development Fund, Qingdao Outstanding Health Professional Development Fund, and Qingdao science and technology program for benefiting the people (19-6-1-59-nsh).

## Conflict of Interest

The authors declare that the research was conducted in the absence of any commercial or financial relationships that could be construed as a potential conflict of interest.

## Publisher’s Note

All claims expressed in this article are solely those of the authors and do not necessarily represent those of their affiliated organizations, or those of the publisher, the editors and the reviewers. Any product that may be evaluated in this article, or claim that may be made by its manufacturer, is not guaranteed or endorsed by the publisher.
